# Anthocyanins abrogate glutamate-induced AMPK activation, oxidative stress, neuroinflammation, and neurodegeneration in postnatal rat brain

**DOI:** 10.1186/s12974-016-0752-y

**Published:** 2016-11-08

**Authors:** Shahid Ali Shah, Faiz Ul Amin, Mehtab Khan, Muhammad Noman Abid, Shafiq Ur Rehman, Tae Hyun Kim, Min Woo Kim, Myeong Ok Kim

**Affiliations:** Neuroscience Pioneer Research Center, Department of Biology and Applied Life Science (BK21), College of Natural Sciences, Gyeongsang National University, Jinju, 660-701 Republic of Korea

**Keywords:** Anthocyanins, Glutamate, AMPK, ROS, Neurotoxicity, Nrf2

## Abstract

**Background:**

Glutamate-induced excitotoxicity, oxidative damage, and neuroinflammation are believed to play an important role in the development of a number of CNS disorders. We recently reported that a high dose of glutamate could induce AMPK-mediated neurodegeneration in the postnatal day 7 (PND7) rat brain. Yet, the mechanism of glutamate-induced oxidative stress and neuroinflammation in the postnatal brain is not well understood. Here, we report for the first time the mechanism of glutamate-induced oxidative damage, neuroinflammation, and neuroprotection by polyphenolic anthocyanins in PND7.

**Methods:**

PND7 rat brains, SH-SY5Y, and BV2 cells treated either alone with glutamate or in combination with anthocyanins and compound C were examined with Western blot and immunofluorescence techniques. Additionally, reactive oxygen species (ROS) assay and other ELISA kit assays were employed to know the therapeutic efficacy of anthocyanins against glutamate.

**Results:**

A single injection of glutamate to developing rats significantly increased brain glutamate levels, activated and phosphorylated AMPK induction, and inhibited nuclear factor-E2-related factor 2 (Nrf2) after 2, 3, and 4 h in a time-dependent manner. In contrast, anthocyanin co-treatment significantly reduced glutamate-induced AMPK induction, ROS production, neuroinflammation, and neurodegeneration in the developing rat brain. Most importantly, anthocyanins increased glutathione (GSH and GSSG) levels and stimulated the endogenous antioxidant system, including Nrf2 and heme oxygenase-1 (HO-1), against glutamate-induced oxidative stress. Interestingly, blocking AMPK with compound C in young rats abolished glutamate-induced neurotoxicity. Similarly, all these experiments were replicated in SH-SY5Y cells by silencing AMPK with siRNA, which suggests that AMPK is the key mediator in glutamate-induced neurotoxicity.

**Conclusions:**

Here, we report for the first time that anthocyanins can potentially decrease glutamate-induced neurotoxicity in young rats. Our work demonstrates that glutamate is toxic to the developing rat brain and that anthocyanins can minimize the severity of glutamate-induced neurotoxicity in an AMPK-dependent manner.

**Electronic supplementary material:**

The online version of this article (doi:10.1186/s12974-016-0752-y) contains supplementary material, which is available to authorized users.

## Background

The central nervous system (CNS) is comprised of several neurotransmitter receptors, with glutamate receptors being one of the major excitatory neurotransmitter receptors that have multiple functions, such as neuronal plasticity, outgrowth, and survival, as well as memory, learning, and behavior [1]. Conversely, glutamate release is under the control of various glutamate transporters as exposure to elevated levels is harmful to neurons. The overstimulation of postsynaptic glutamate receptors causes neuronal injury/death, which is termed as glutamate excitotoxicity as is believed to be involved in amyotrophic lateral sclerosis (ALS) and several other CNS disorders [2–4]. This glutamate excitotoxicity is believed to arise specifically from the entrance of a high rate of Ca^2+^ into the neurons as a result of over-stimulated postsynaptic glutamate receptors [5]. The excess amount of intracellular Ca^2+^ levels can increase the burden of mitochondrial Ca^2+^, thereby inducing mitochondrial damage and reactive oxygen species (ROS) generation in the mitochondria [6–8]. Overproduction of ROS is thought to cause a diversity of diseases. There is a sophisticated antioxidant defense mechanism in cells that aides in coping with ROS levels under normal physiological conditions, but under certain conditions, such as excessive ROS and inflammation, excessive Ca^2+^ can cause cellular dysfunction and remodeling [9, 10].

NF-E2-related factor 2 (Nrf2) is a transcription factor that is bound to the antioxidant response element (ARE) and can induce the regulation of various antioxidant encoding genes, particularly heme oxygenase-1 (HO-1) [11]. Previous reports have proven that the activation of Nrf2 in cells and tissues in response to oxidative stress protects against oxidative injury. Under normal conditions, Nrf2 is localized in the cytoplasm [12], while under oxidative stress conditions, it translocates into the nucleus and transactivates its target genes through ARE. Various protein kinases have been reported to be involved in the ARE-mediated gene Nrf2 activation in response to the oxidative stress signals including 5′ AMP-activated protein kinase (AMPK) [13]. AMPK primarily functions as an energy sensor [14]. AMPK activation not only suppresses ATP-consuming metabolic pathways but also accelerates the energy-producing signaling pathways to offer cellular protection against any stress.

Natural polyphenolic compounds that are obtained from fruits and vegetables have received a great deal of interest during the last decade, due to their potential to inhibit oxidative stress, as reported in various studies [15]. Anthocyanins are a group of natural phenolic compounds that confer the different colors of plants and fruits. Evidence suggests that anthocyanins are strong natural antioxidants [16, 17]. Our research group has recently reported the anti-apoptotic, antioxidant, and anti-obesity effects of Korean black bean-derived anthocyanins in different experimental models [18–22].

In the current study, we extended our line of investigation to elucidate the exact mechanism of the neuroprotection of Korean black bean-derived anthocyanins against glutamate-induced excitotoxicity, oxidative stress, neuroinflammation, and neurodegeneration in the hippocampus of the developing rat brain and in SH-SY5Y and BV2 cells.

## Methods

### Animals and drug treatment

Sprague-Dawley (SD) rat pups (18-g average body weight) on postnatal day 7 (*n* = 5 rats/group) were randomly divided into the following eight groups (the treatment outlines are given in Additional file 1):

1. Control (C)
2. Glutamate for 2 h (Glu 2 h)
3. Glutamate for 3 h (Glu 3 h)
4. Glutamate for 4 h (Glu 4 h)
5. Glutamate for 4 h + anthocyanins (Glu 4 h + Anth)
6. Glutamate for 4 h + compound C (Glu 4 h + CC)
7. Glutamate for 4 h + compound C + anthocyanins (Glu 4 h + CC + Anth)
8. Anthocyanins (Anth)

Glutamate (10 mg/kg), anthocyanins (100 mg/kg), and compound C (10 mg/kg) [23] in saline solution were intraperitoneally (i.p.) injected. Glutamate was administered for 2, 3, or 4 h. The control animals received 0.9 % saline solution, and all the rats were decapitated after 2, 3, or 4–12 h. All the experimental procedures were approved by local ethical committee for animals of the Department of Biology, Division of Applied Life Sciences, Gyeongsang National University South Korea.

### Cell culturing and drug treatment

Murine BV2 microglia and human neuroblastoma SH-SY5Y cells were maintained in 10 % FBS- and 1 % penicillin/streptomycin-supplemented DMEM (Dulbecco’s modified Eagle’s medium) medium in a humidified 5 % CO_2_ incubator at 37 °C. The cells were treated with glutamate (30 mM), glutamate plus anthocyanins (30 mM + 20 μg/ml), glutamate plus AMPK siRNA (30 mM + 200 nM), glutamate plus AMPK siRNA plus anthocyanins (30 mM + 200 nM + 20 μg/ml), glutamate plus compound C (30 mM + 20 μM), and glutamate plus compound C and anthocyanins (30 mM + 20 μM + 20 μg/ml) for 3 h.

### AMPK gene silencing

Small interfering RNA (siRNA) was purchased from Santa Cruz Biotechnology (sc-45312). Cultured SH-SY5Y cells were transfected with 200 nM siRNA using Lipofectamine 2000 reagent (Invitrogen) for 24 h [24], then the DMEM medium was replaced with glutamate and anthocyanins as indicated in the respective “Methods” section.

### Cell viability assay

The MTT (3-(4,5-dimethylthiazol-2-yl)-2,5-diphenyltetrazolium bromide) assay was performed according to the manufacturer’s instructions (Sigma) to assess the SH-SY5Y cell viability after treatment. The cells were cultured in 96-well plates at a density of 1 × 10^4^ cells per well containing 100 μl DMEM. When the cells were attached after 24 h, the medium was refreshed with the indicated concentration of glutamate (10, 20, and 30 mM) and anthocyanins (10, 20, and 30 μg/ml), while control cells received only DMEM medium. The cells were then incubated for an additional 3 h. After being cultured for 3 h, the cells were incubated with MTT solution for another 4 h at 37 °C. Subsequently, the medium was replaced with DMSO in each well. Finally, the absorbance was measured at 570 nm. All experiments were performed independently in triplicate.

### Western blot analysis

Western blot analysis details were conducted as previously performed in our lab [25]. Briefly, the animals were euthanized after 4 h following treatment of glutamate with or without anthocyanins. Then, the brains were carefully (hippocampus) collected and placed on dry ice for freezing tissue. Similarly, after treatment, the SH-SY5Y and BV2 cells were collected in PBS and centrifuged, and the supernatant was removed. The remaining pellet was dissolved in Pro Prep Protein Extraction Solution, according to the manufacturer’s protocol (iNtRON Biotechnology) to make cell lysates. The brain homogenates and cell lysates were quantified with Bio-Rad protein assay solution. The homogenates (20 μg protein) were fractionated by SDS-PAGE on 4–12 % (Bolt™ Mini Gels, Life Technologies). After transfer, membranes were blocked in 5 % skim milk (or BSA) and incubated overnight at 4 °C with primary antibody, and cross-reacting proteins were detected by ECL after reaction with horseradish peroxidase-conjugated secondary antibodies. The primary antibodies (1: 500 in Tris-buffered saline with Tween (TBST)) included rabbit-derived anti-COX2, anti-TNFα, anti-p-AMPKTh^172^, anti-AMPK, anti-Nrf2, anti-caspase-3, anti-iNOS, anti-p-NF-*k*B, mouse-derived anti-β-actin, anti-GFAP, anti-heme oxygenase-1 (HO-1), and goat-derived anti-Iba-1 from Santa Cruz Biotechnology (Santa Cruz, CA, USA). After using membrane-derived secondary antibodies (1: 1000 in TBST), ECL (Amersham Pharmacia Biotech, Uppsala, Sweden) detection reagent was used for visualization according to the manufacturer’s instructions. Densitometry analysis of the bands was performed using Sigma Gel software (SPSS, Chicago, IL, USA). Density values were calculated in arbitrary units (A.U.) relative to the untreated control.

### Tissue collection and sample preparation

The animals were euthanized after 12 h of drug treatment to conduct morphological studies as we reported earlier [26]. Briefly, the brain tissues from all the treated groups after 12 h were subjected to transcardial perfusion with 4 % ice-cold paraformaldehyde. After postfixing these brain tissues, 4 % paraformaldehyde was transferred to 20 % sucrose. The tissues were frozen in OCT (Tissue-Tek O.C.T. Compound Medium, Sakura Finetek USA, Inc., Torrance, CA, USA), sectioned into 14–16-μm sections in the coronal plane with a CM 3050S cryostat (Leica, Wetzlar, Germany). The sections were thaw-mounted on Probe-On positively charged slides (Thermo Fisher Scientific Inc., Waltham, MA, USA).

### Fluoro-Jade B staining

Fluoro-Jade B staining was performed as reported earlier [27]. Chamber slides were air-dried overnight. The slides were kept in a solution of 80 % ethanol and 1 % sodium hydroxide then in 70 % alcohol for 5 and 2 min, respectively, followed by immersion in distilled water for 2 min. The slides were placed for 10 min in 0.06 % potassium permanganate solution. The slides were then rinsed with distilled water and immersed in a solution containing 0.1 % acetic acid and 0.01 % Fluoro-Jade B for 20 min. After rinsing with distilled water and applying DAPI, the slides were dried, and glass cover slips were mounted on slides with mounting medium. Images were captured using an FITC filter on a confocal laser scanning microscope (FV 1000, Olympus, Japan).

### Immunofluorescence

Immunofluorescence stainings were performed as we previously reported [19]. Shortly, the slides were washed with 1× PBS and incubated with proteinase K solution at room temperature. After blocking in normal goat/rabbit serum, primary antibodies (1: 100 in PBS, mentioned in the “Western blotting” section) were applied overnight at 4 °C. Fluorescence-based (FITC and TRITC from Santa Cruz Biotechnology) secondary antibodies (in PBS) were applied at room temperature. DAPI was used to stain the nucleus. The slides were mounted with glass coverslips, and the images were taken using a confocal microscope (FluoView FV 1000; Olympus, Tokyo, Japan).

### TUNEL staining

To determine apoptotic cell death, TUNEL (terminal deoxynucleotidyl transferase (TdT)-mediated dUTP nick-end labeling) staining was performed according to the manufacturer’s recommendations. An in situ cell death detection kit was purchased from Roche (Cat. No. 11684809910).

### Oxidative stress (ROS) detection in vivo and in vitro

The ROS quantification assay in the brain homogenates of all treated groups was conducted as reported earlier in the literature and by our research group [28, 29] with slight modification. Briefly, the brain homogenates from the respective groups were diluted 1:20 times with Locke’s buffer (ice-cold) to get 5 mg tissue/ml concentration. Then, the reaction mixture (1 ml) having Locke’s buffer of pH 7.4, 0.2 ml brain homogenate, and 10 ml of DCFH-DA (5 mM) was incubated for 15 min at room temperature to allow the DCFH-DA to be incorporated into any membrane-bound vesicles and the diacetate group cleaved by esterases. After 30 min of further incubation, the conversion of DCFH-DA to the fluorescent product DCF was measured using a spectrofluorometer with excitation at 484 nm and emission at 530 nm. ROS formation was quantified from a DCF-standard curve and data are expressed as pmol DCF formed/min/mg protein. Similarly, an in vitro ROS assay was performed with slight modification as previously described [26]. Briefly; SH-SY5Y cells were sub-cultured in 96-well plates in 200 μl DMEM that was supplemented with 10 % FBS and 1 % penicillin/streptomycin in every well. The cells were incubated for 24 h at 37 °C in a humidified incubator having 5 % CO_2_. The next day, the media was replaced by fresh media that contained Glu (30 mM), Glu plus anthocyanins (30 mM + 20 μg/ml), Glu plus compound C (30 mM + 20 μM), or Glu plus compound C and anthocyanins (30 mM + 20 μM + 20 μg/ml) for an additional 30 min. DCFDA (2′,7′-dichlorofluorescin diacetate) 600 μM dissolved in DMSO/PBS was added to each well and incubated for 30 min. The plates were then read in ApoTox-Glo™ (Promega) at 488/530 nm.

### Glutamate assay

Glutamate assay kit (Cat. # KA1670) from Abnova (Taipei, Taiwan) was used to quantify glutamate in hippocampal brain tissue homogenates according to the manufacturer’s instruction.

### Enzyme assays

The hippocampal rat brain homogenates and SH-SY5Y cell lysates of the experimental groups were evaluated using ELISA assays for total NF-_*k*_Bp65 (Life Technologies, Catalog #KHO0371) and Cyclex AMPK (Enzo Life Sciences), as per the manufacturer’s recommended protocols.

### COX2 assay

An ELISA assay for COX2 (R&D Systems, Inc. 614 McKinley Place NE Minneapolis, MN 55413, USA) was performed. BV2 and SH-SY5Y cells were sub-cultured in 96-well plates, and treatment was performed in different groups, as described earlier, and then fixed with 4 % paraformaldehyde, as per the manufacturer’s recommendations.

### GSH assays

The levels of total GSH and GSH/GSSG ratio were determined by using glutathione assay kit obtained from BioVision (BioVision Incorporated155 S. Milpitas Boulevard, Milpitas, CA 95035, USA), Fluorometric Assay Kit (Catalog #K264-100), according to the manufacturer’s instructions.

### Statistical analysis

A computer-based Sigma Gel System (SPSS Inc., Chicago, IL) and the ImageJ program were used to analyze the density and integral optical density (IOD) of scanned X-ray films of Western blot and immunofluorescence images. Density values were expressed as the mean ± SEM. All data are presented as the mean ± SEM. A one-way ANOVA followed by Student’s *t* test (non-parametric Mann-Whitney and Wilcoxon tests) were used to determine the statistical significance (*P* < 0.05) of the obtained data.

## Results

### Exogenous glutamate induced multiple neurotoxic effects in the developing rat brain in a time-dependent manner

**Fig. 1:**
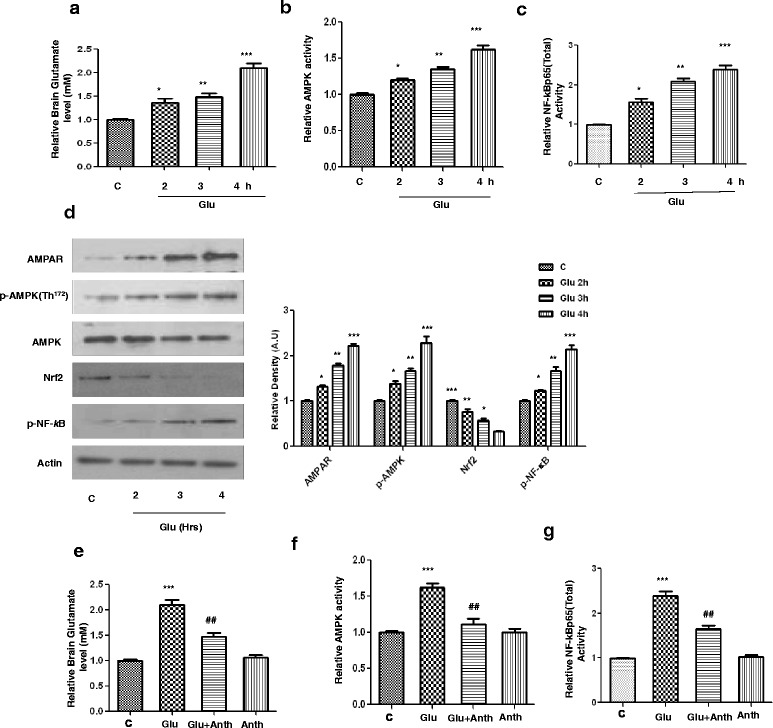
Glutamate increased brain glutamate level, AMPK activation, and Nrf2 inhibition in a time-dependent manner in the hippocampal CA1 region of the developing rodent brain. **a** Brain glutamate level, **b** AMPK activity, and **c** total NF-_*k*_Bp65 activity analysis using ELISA histograms in the CA1 region brain homogenates after glutamate administration in a time-dependent manner. **d** The immunoblot protein expressions of AMPAR, phospho-AMPK, total AMPK, Nrf2, and β-actin in the developing rat brain homogenates 2, 3, and 4 h following glutamate administration. Relative density histograms depict the differences across the groups. Representative blots obtained in sections were prepared from at least five animals per group. Relative histograms of **e** brain glutamate level, **f** AMPK activity, and **g** total NF-_*k*_Bp65 in the CA1 region brain homogenates of experimental groups, including glutamate and anthocyanins with or without glutamate treated P7 rats after 4 h. These assays were conducted in triplicate with similar results according to the manufacturer instructions. Significance, *P < 0.05, **P < 0.01, and ***P < 0.001 and ^##^ *P* < 0.01, respectively

The brain glutamate level was analyzed with a glutamate assay kit method in developing brain homogenates after exogenous glutamate administration. Our results (Fig. 1a) indicated that exogenous glutamate produced significant up-regulation of brain glutamate in the hippocampus of young rats after 2, 3, and 4 h (where 4 h was the most significant increase in glutamate level) following its administration in a time-dependent manner. Similarly, glutamate also induced the activities of AMPK and NF-_*k*_Bp65 in the same manner within 2 to 4 h, as assessed with the help of a respective ELISA assay kit method (Fig. 1b, c). We then examined the protein expression level of AMPAR, phosphorylated AMPK^Th172^ (p-AMPK), Nrf2, and phosphorylated nuclear factor kappa (p-NF-_*k*_B) in the hippocampal brain homogenates of the developing brain in a time-dependent manner. The results (Fig. 1d) indicate that exogenous glutamate administered to the developing rats significantly increased the expression level of AMPARs, p-AMPK, and p-NF-_*k*_B after 2, 3, and 4 h in the hippocampus (whereas after 5 h, glutamate level and its toxic effects began to decline and that data is not shown here). Similarly, this glutamate also caused the inhibition of hippocampal Nrf2 proteins in a time-dependent manner (Fig. 1d).

### Anthocyanins are beneficial against glutamate-induced neurotoxicity in the developing brain

**Fig. 2:**
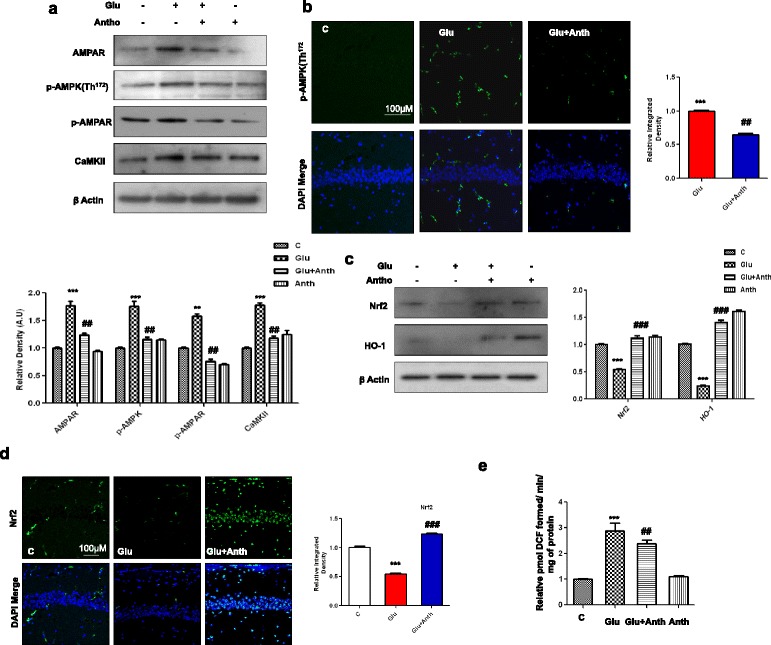
Beneficial effects of anthocyanins on glutamate-induced excitotoxicity in the developing rodent brain. **a** The immunoblots of AMPAR, p-AMPK, p-AMPA, and CaMKII proteins along with their relative density histograms in the experimental groups. β-Actin was used as a loading control. **b** The immunostaining images and respective relative IOD histogram of p-AMPK protein in the CA1 region of young rats that were treated with glutamate with or without anthocyanins. The images show p-AMPK (*green*) and DAPI (*blue*) color. **c** The Western blot results of Nrf2 and HO-1 proteins in the brain homogenates of P7 rats. The significant differences among the different treated groups are indicated by relative density histograms. The bands were quantified with sigma gel software and density histograms (expressed in arbitrary units, i.e., A.U.) relative to control were made with Prism GraphPad. **d** The immunofluorescence investigation of Nrf2 in the CA1 section of developing rats after glutamate and anthocyanin administration. The tissues were post-fixed in 4 % paraformaldehyde and 20 % sucrose solution for 72 h. The immunofluorescence staining techniques are given in the “Methods” section. **e** The histogram indicates the relative ROS contents in the different experimental groups. CA1 rat brain homogenates were prepared according to the protocol of the ROS assay, as cited in the manuscript. The assay was repeated for three times with the same results. Significance, **P < 0.01 and ***P < 0.001 and ^##^ *P* < 0.01 and ^###^ *P* < 0.001, respectively

We then evaluated the therapeutic efficacy of anthocyanins against glutamate-induced neurotoxicity in the developing rat brain. As shown above, it was observed that exogenous glutamate could cause a maximal up-regulation of hippocampal brain glutamate levels as well as AMPK and NF-_*k*_Bp65 activities 4 h after its administration. For this reason, we co-treated rat pups with anthocyanins along with glutamate. Anthocyanin treatment significantly reduced brain hippocampal glutamate levels as well as AMPK and NF-_*k*_B activities against exogenously injected glutamate (Fig. 1e–g). Similarly, Western blot results revealed that anthocyanin administration also significantly down-regulated glutamate-induced AMPA receptor expression and p-AMPK activation in the developing rat brain (Fig. 2a). Immunofluorescence investigation of p-AMPK also supported our Western blot results that indicated that anthocyanins could reduce glutamate-induced AMPK activation in the hippocampal CA1 region (Fig. 2b). Additionally, the exogenously administered glutamate also increased the intracellular calcium abundance by upregulating the expression level of CaMKII protein, which was accompanied by AMPA receptor phosphorylation at Ser^845^ (i.e., p-AMPA Ser^845^); however, the rat pups that received anthocyanins as a co-treatment significantly inhibited the glutamate-induced up-regulation of CaMKII and p-AMPA Ser^845^ protein expression, as shown in Fig. 2a.

### Anthocyanins increased cellular glutathione levels and activated the Nrf2/HO-1 signaling pathway against glutamate-induced oxidative stress in the developing brain

To examine the antioxidant effect of anthocyanins, we first analyzed glutathione (GSH and GSSG) levels accompanied by the quantification of the expression of Nrf2 protein and its downstream signaling gene heme oxygenase-1 (HO-1) with immunoblotting. Our results show that glutamate treatment after 4 h significantly reduced cellular GSH content and GSH/GSSG ratio compared with the saline-treated control in the developing rat brain (Additional file 1: Fig. S1). Anthocyanins treatment significantly increased cellular GSH content and GSH/GSSG ratio against glutamate (Additional file 1: Fig. S1). Similarly, we have already shown that exogenously administered glutamate can significantly suppress Nrf2 after 4 h (Fig. 1d). For this reason, the rat pups that received anthocyanins with or without glutamate were euthanized after 4 h and their brain homogenates were subjected to Western blotting and a ROS assay. The Western blot results revealed that anthocyanin treatment significantly increased the expression of Nrf2 and HO-1 proteins in the developing brain. Additionally, the immunofluorescence examination of Nrf2 protein in brain tissue agreed with our immunoblot results and showed that anthocyanin treatment increased the expression of Nrf2 (in the nucleus, as shown Additional file 1) in the hippocampal CA1 region of the developing rat brain (Fig. 2c, d, the high magnification expanded image is given in Additional file 1). Glutamate has been reported for its ability to produce ROS. For this reason, the ROS assay was conducted with the brain homogenates of the experimental groups. The assay results indicated that anthocyanin treatment significantly inhibited glutamate-induced ROS production in the developing rat brain (Fig. 2e).

### Anthocyanins attenuated glutamate-induced neuroinflammation and neurodegeneration

**Fig. 3:**
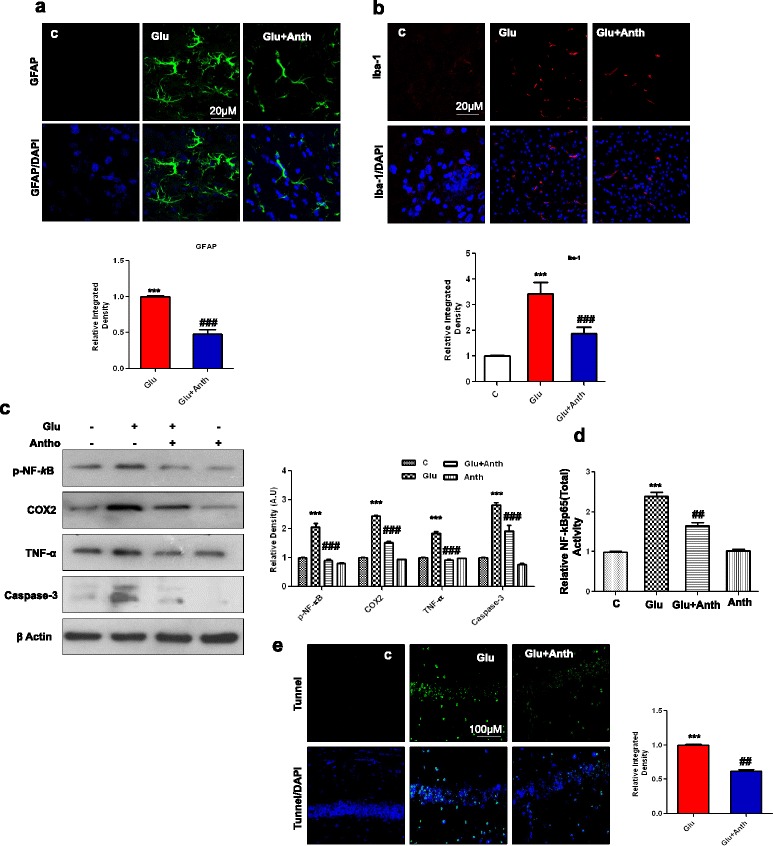
Anthocyanins reduced glutamate-induced glial cell activation, neuroinflammation, and DNA damage in hippocampal CA1 region of the developing rat brain. Given are representative immunofluorescence images along with relative IOD histograms of **a** astrocytes (GFAP)-positive cells and **b** microglia (Iba-1)-positive cells in the CA1 region of experimental groups. Images represent immunostaining performed with tissue sections prepared from at least five rats in each group. Panels representing hippocampal CA1 region immunostained with GFAP (*green*), Iba-1(*red*) counterstained with DAPI (blue) of young rat brain. **c** The immunoblot represents proinflammatory markers, including p-NF-_*k*_B, COX2 and TNF-α proteins, which are accompanied by their relative density histograms. **d** The ELISA histogram of NF-_*k*_Bp65 (total) in the brain homogenates of treated animals. The assay was repeated three times according to the manufacturer’s instruction. **e** The extent of DNA damage in the CA1 region by glutamate was analyzed by conducting TUNEL assay. To quantify DNA damage, ImageJ and Prism GraphPad programs were used. The values represent the mean ± SEM for the indicated proteins (*n* = 5 animals per group). Significance, ***P < 0.001 and ^##^ *P* < 0.01 and ^###^ *P* <0.001, respectively

The extent of neuroinflammation that was induced by glutamate was assessed with immunofluorescence, Western blot, and ELISA assays. A single episode of glutamate injection caused significant induction of glial cell activation, including both astrocytes (GFAP) and microglia (Iba-1) (Fig. 3a, b), which was accompanied by upregulated proinflammatory p-NF-_*k*_B and its downstream signaling molecules, such as cyclooxygenase-2 (COX-2), tumor necrosis factor-ά (TNF-ά), and caspase-3 protein expressions in the hippocampal CA1 region of the developing rat brain (Fig. 3c). Anthocyanin supplementation significantly attenuated not only glutamate-induced glial cells activation but also markedly inhibited p-NF-_*k*_B, COX-2, and caspase-3 protein expression, as assessed with immunofluorescence and Western blotting methods. The activity histogram of NF-_*k*_Bp65 (total), which was examined with the ELISA methods, is also in agreement with the immunoblot results that anthocyanins can inhibit p-NF-_*k*_B activity in the developing brain (Fig. 3d). Additionally, glutamate-induced neurodegeneration in the immature rodent brain was assessed with TUNEL staining. The results revealed that glutamate was able to induce significant DNA fragmentation in the hippocampal CA1 region of the developing rodent brain. In contrast, anthocyanin treatment significantly decreased glutamate-induced DNA fragmentation in the CA1 region of the P7 rodent brain (Fig. 3e).

### Glutamate-induced neurotoxicity is AMPK dependent in the developing rat brain

**Fig. 4:**
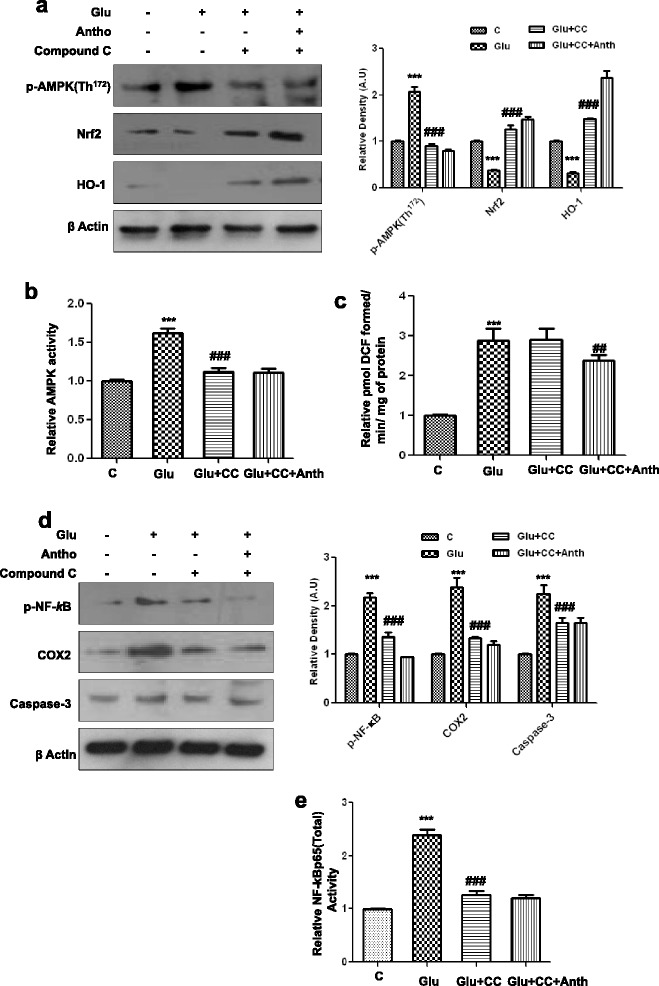
Glutamate-induced neurotoxicity in the developing rodent brain is AMPK dependent. **a** The expression level of p-AMPK, Nrf2, and HO-1 proteins in the P7 rat brain homogenates that were treated with glutamate or glutamate with or without compound C and anthocyanins for 4 h. Their densities were measured with the help of Sigma Gel software. Their respective relative density histograms were made with Prism GraphPad. The membranes were redeveloped for β-actin and used as a loading control. **b**, **c** The histograms of AMPK activity and ROS assay were conducted with brain homogenates of the abovementioned experimental animal groups. The assays were performed in triplicate with the same results. **d** The Western blot analysis of p-NF-_*k*_B, COX2, and caspase-3 proteins in the hippocampus of postnatal day 7 rat brain following treatment with glutamate, glutamate and compound C, or glutamate, compound C, and anthocyanins. The relative integrated density for the abovementioned proteins are depicted in the histograms. The density values are expressed in arbitrary units as the mean ± SEM for the indicated proteins (*n* = 5 animals per group). Details are provided in the “Methods” section. **e** The activity histogram of NF-_*k*_Bp65 (total) in the brain homogenates of treated animal’s measured with ELISA kit method. Significance; ***P < 0.001 and ^###^ *P* < 0.001, respectively

To determine whether glutamate-induced neurotoxicity is AMPK dependent, we injected compound C, an inhibitor of AMPK, along with glutamate or without anthocyanins into immature rats. Interestingly, compound C administration significantly reduced glutamate-induced AMPK activation, as shown in Fig. 4a, b. These results show that compound C after 4 h blocked AMPK protein expression (Fig. 4a) as well as activity (Fig. 4b), as indicated by Western blot and ELISA assay, respectively. Accordingly, the ROS assay that was conducted also supports the notion that glutamate-induced ROS can activate AMPK, as compound C has no effect on the ability of either glutamate-induced oxidative stress or anthocyanins to reduce ROS in the developing brain (Fig. 4c). We measured Nrf2 and HO-1 proteins levels using Western blot analysis. The immunoblot quantification revealed that compound C significantly blocked the ability of glutamate to suppress Nrf2 and HO-1 in the developing rat brain (Fig. 4d). Similarly, compound C also blocked glutamate from significantly activating p-NF-_*k*_B (both its activity and expression), COX2, and caspase-3 proteins in the hippocampus of the young rat brain (Fig. 4d, e). Interestingly, compound C treatment affected the ability of anthocyanins to up-regulate Nrf2, HO-1, p-NF-_*k*_B, COX2, and caspase-3 proteins (Fig. 4a, d, e). This suggests that in the postnatal rat brain, glutamate produces its neurotoxicity by activating AMPK.

### Anthocyanins enhanced cell viability and decreased glutamate-induced neurodegeneration in vitro

**Fig. 5:**
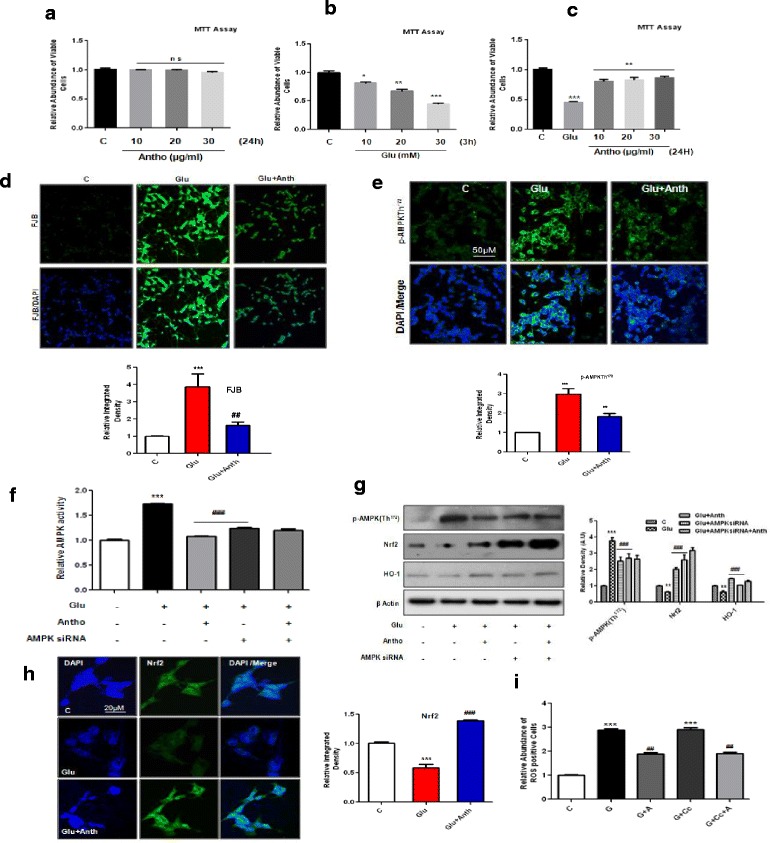
The beneficial effects of anthocyanins against glutamate-induced toxicity in SH-SY5Y cells. **a**–**c** The cell viability histograms of SH-SY5Y cells, as indicated by the MTT assay. **a** The cells were incubated with three different (indicated) concentrations of anthocyanins for 24 h. **b** The cell viability histogram of SH-SY5Y cells that were treated with the indicated concentrations of glutamate for 3 h. **c** The cell viability histogram after pretreating cells (12 h) with three concentrations of anthocyanins and glutamate (30 mM) for additional 3 h. All details are provided in the “Methods” section. **d** The immunofluorescence images and respective relative IOD histogram of FJB (green)-positive and DAPI (blue) SH-SY5Y cells. Cells were grown in four-well chamber slides and then treated with the mentioned (“Methods” section) concentrations of glutamate and anthocyanins. The FJB staining was repeated in triplicate. **e** The immunostained images of p-AMPK (*green*) protein counterstained with DAPI (*blue*) in the SH-SY5Y cells. The *panels* represent different treating groups such as untreated control, glutamate, or glutamate and anthocyanins. **f** The histogram of relative AMPK activity assay conducted with cell lysate experimental treating groups having siRNA of AMPK. **g** The Western blot analysis of p-AMPK, Nrf2, and HO-1 protein expressions and their relative density histograms after treating cells with glutamate and glutamate and anthocyanins and in the presence or absence of AMPK siRNA. **h** The immunostaining images and respective relative IOD histograms of Nrf2 protein in SH-SY5Y cells. **i** The ROS assay histogram after treating SH-SY5Y cells with glutamate or glutamate and anthocyanins with or without compound C. The groups and other experimental details are provided in the “Methods” section. Significance, *P < 0.05, **P < 0.01, ***P < 0.001 and ^##^ *P* < 0.01 and ^###^ *P* <0.001, respectively

The MTT assay was conducted to measure the cell viability of SH-SY5Y cells after treatment with different concentrations of glutamate and anthocyanins. The results show that anthocyanins in three different concentrations (i.e., 10, 20, and 30 μg/ml) were non-toxic to cells (Fig. 5a), in contrast to that of glutamate in three concentrations (i.e., 10, 20, and 30 mM), which significantly reduced SH-SY5Y cell viability (Fig. 5b). Similarly, all three different concentrations of anthocyanins significantly increased SH-SY5Y cell viability against glutamate (30 mM), as shown in Fig. 5c. Additionally, the extent of glutamate-induced neuronal loss was measured through Fluoro-Jade B (FJB) staining in SH-SY5Y cells. The FJB results indicated that glutamate (30 mM) significantly induced neuronal apoptosis in SH-SY5Y cells, as shown in Fig. 5d. Similarly, these images also revealed that anthocyanins (20 μg/ml) reduced the extent of neuronal apoptosis in vitro (Fig. 5d).

### Glutamate-induced neurotoxicity in vitro is AMPK dependent

The mechanism of action of glutamate in vitro was confirmed by conducting experiments with SH-SY5Y and BV2 cells. We first silenced AMPK with its siRNA and then analyzed the expression levels of different proteins. The results showed that the anthocyanin treatment significantly reduced p-AMPK immunoreactivity (Fig. 5e) and AMPK activity (Fig. 5f) against glutamate in SH-SY5Y cells. Similarly, anthocyanin treatment also reduced glutamate-increased p-AMPK protein expression, as shown in Fig. 5g. Additionally, anthocyanins enhanced Nrf2 and HO-1 expression, which are antagonistic towards the effects of excessive glutamate, in vitro (Fig. 5g). The expressions levels of Nrf2 were measured both with immunoblotting and immunofluorescence methods (Fig. 5g, h). AMPK was silenced with its siRNA, which not only decreased p-AMPK activity but also reduced its expression (Fig. 5f, g). Interestingly, the effect of anthocyanins on the expressions level of p-AMPK, Nrf2, and HO-1 was also observed following siRNA-mediated AMPK silencing (Fig. 5f). Similarly, the ROS assay that was conducted revealed that anthocyanins significantly inhibited glutamate-induced ROS production in cells. Again, compound C was observed to have no effect on ROS generation, thereby supporting our in vivo results that this ROS is independent of AMPK in vitro as well (Fig. 5i).

**Fig. 6:**
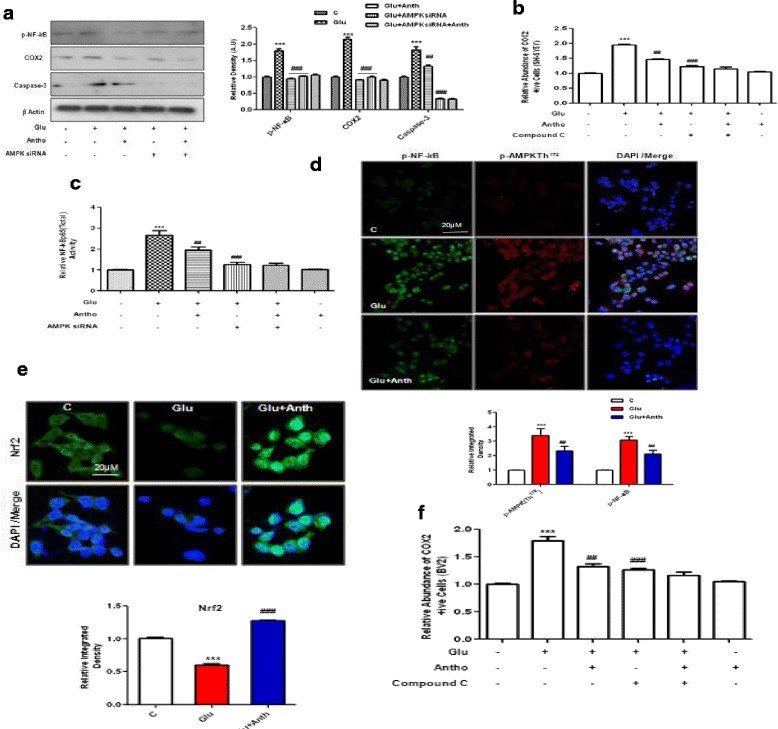
Glutamate-induced toxicity in SH-SY5Y and BV2 cells is AMPK dependent. **a** The Western blot analysis and relative integrated density histograms of p-NF-_*k*_B, COX2 and caspase-3 proteins in SH-SY5Y cells. The treatment details are the same as described in the “Methods” section. β-Actin was used as a loading control. **b** The activity histogram of NF-_*k*_Bp65 (total) in the cell lysates, as measured with an ELISA kit. **c** The COX2 assay was performed with SH-SY5Y cells after treatment with the indicated concentrations of glutamate, anthocyanins, and compound C for the indicated duration of time. After treatment, cells were fixed prior to performing the assay as per the manufacturer’s instructions. The immunostaining images (**d**) of p-NF-_*k*_B (*green*), p-AMPK (*red*) (**e**), Nrf2 (*green*) and DAPI (*blue*) in the BV2 cells treated with glutamate and anthocyanins. Their densities are depicted in the relative IOD histogram. **f** The COX2 assay histogram in BV2 cells was conducted according to the manufacturer’s instructions. Significance, ***P < 0.001 and ^##^ *P* < 0.01, ^###^ *P* < 0.001, respectively

We then measured the expression level of p-NF-_*k*_B, COX2 and caspase-3 after treating with glutamate and anthocyanins with or without AMPK siRNA-treated SH-SY5Y cells. Again, anthocyanins significantly reduced the expression level of p-NF-_*k*_B, COX2 and caspase-3; however, AMPK knock-down completely blocked the ability of glutamate to up-regulate the abovementioned proinflammatory and apoptotic marker in SH-SY5Y cells (Fig. 6a). The silencing of AMPK also affected the inhibitory potential of anthocyanins. Similarly, the activities of NF-_*k*_B and COX2, as measured by ELISA kit methods, also supported the notion that glutamate-induced NF-_*k*_B and COX2 activity up-regulation was AMPK dependent (Fig. 6b, c).

**Fig. 7:**
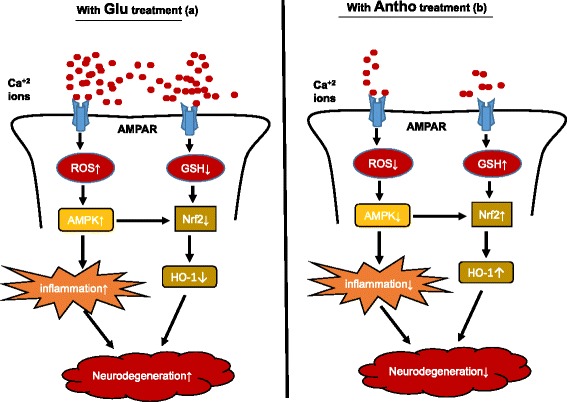
The signaling pathway of anthocyanins neuroprotection against the glutamate-induced neurotoxicity. This scheme depicts the number of various events taking place after the glutamate (**a**) and anthocyanins (**b**) treatments. It is evident from the flow sheet that anthocyanins treatment completely inhibited glutamate-induced neurodegeneration in the developing rats

We also used BV2 cells to examine the beneficial effects of anthocyanins against glutamate-induced cytotoxicity. The double immunofluorescence results indicated that anthocyanins reduced the expression levels of p-AMPK and p-NF-_*k*_B against glutamate in BV2 cells (Fig. 6d). Similarly, anthocyanin treatment enhanced Nrf2 immunoreactivity in the nucleus of BV2 cells (Fig. 6e). Additionally, we analyzed the activity of COX2 by blocking AMPK with compound C in vitro and measuring activity via an ELISA kit*.* The results showed that anthocyanin treatment decreased glutamate-induced COX2 activity (Fig. 6f). Compound C treatment diminished the ability of glutamate to induce an increase in COX2 activity and reduced anthocyanins ability to inhibit COX2 activity in BV2 cells, which indicates that this process is dependent upon AMPK (Fig. 6f). All these events in the current study are highlighted in detail in the Fig. 7.

## Discussion

The current study demonstrates several important findings (as shown in detail in Fig. 7). First, it reports that exogenously administered glutamate in the developing rat can increase brain glutamate levels as well as activate and phosphorylate AMPK and NF-_*k*_B, which is accompanied by Nrf2 protein inhibition. These toxic effects that were induced by glutamate were observed following 2, 3, and 4 h of its administration, as measured and quantified by Western blot and ELISA assays. Those results indicate that glutamate can induce a maximal toxic effect after 4 h when compared to 2 and 3 h. Second, this study provides evidence that after 4 h, anthocyanins can reduce brain glutamate levels, AMPK activation, ROS production, and inflammation. Third, anthocyanins increased glutathione levels and stimulated Nrf2/HO-1 signaling, which has an important role in cellular defense against oxidants and toxic chemicals. Finally, we reported that blocking AMPK either with compound C or with siRNA not only negatively regulates glutamate-induced AMPK activation and inflammation but also can abolish the neuroprotective abilities of anthocyanins in young rats as well as in SH-SY5Y cells.

AMPK and oxidative stress have long been associated with neurodegenerative diseases. Oxidative stress disrupts mitochondrial respiration and damages mitochondria, which ultimately induce apoptotic cell death and degeneration [30]. Mitochondria, a major regulator of apoptosis, have an important role in controlling cell life and death [31]. Current findings indicate that anthocyanins decrease glutamate-induced ROS and energy reduction, thereby protecting against oxidative stress.

Here, for the first time, we found that AMPK signaling mediates a crucial role in the antioxidant activity of anthocyanins against glutamate-induced oxidative stress. This beneficial effect of anthocyanins, both in young rats and in cells, is associated with up-regulation of glutathione (both GSH levels and GSSG ratio) and the stimulation of Nrf2/HO-1 as an important antioxidant-signaling pathway. Our findings demonstrated that anthocyanins both inhibited glutamate-upregulated proinflammatory and neuroapoptotic markers as well as suppressed endogenous antioxidant molecules via activated AMPK reduction. Recently, anthocyanins have been shown to exert AMPK-mediated neuroprotection against kainic acid-induced excitotoxicity in cells [22]. Anthocyanins are comprised of various flavonoids that have been reported to have beneficial effects against oxidative stress, inflammation, and neurodegeneration [32–34]. These beneficial effects of anthocyanins are due to their antioxidative characteristics, as demonstrated by several researchers in their reports [16, 17]. Similarly, numerous studies have indicated that cyanidin-3-O-glucoside (C3G), one of the active components of anthocyanins, is a potent neuroprotective agent against cerebral ischemia and β-amyloid-induced mitochondrial damage [35, 36] and can block the release of apoptosis-inducing factor (AIF) in focal cerebral ischemia [37]. The blueberry-enriched diet has been shown to reduce kainic acid-induced oxidative stress and excitotoxicity mediated memory impairment [38].

Oxidative stress is one of the earliest pathological changes in neurodegenerative diseases such as Alzheimer’s disease (AD). The hippocampus and cortex sections of the brain are vulnerable to oxidative stress and are associated with the development of cognitive impairment as a feature of sporadic AD. Similarly, another risk factor for AD is aging, in which the endogenous system is unable to scavenge free radicals and ultimately produces oxidative stress in the brain [39]. Additionally, the brain has a relatively weak antioxidant defense system compared to other organs of the body and so the activation of the endogenous antioxidant system is particularly vital for those tissues. In this regard, the stimulation of Nrf2-ARE signaling pathways in the brain has been considered as one of the major pharmaceutical strategies for the treatment and prevention of neurodegenerative disease and brain aging. Nrf2 is believed to be involved in regulating phase II antioxidant responses, which induce the activation of several free radical scavengers and beneficial enzymes. HO-1 is one of the important genes in the brain and is activated by Nrf2, as demonstrated by intensive studies that have a crucial role in providing shelter to neurons against cell death. Accordingly, numerous reports have described that Nrf2 activation [40, 41] induces an increase in HO-1 in response to several neurodegenerative disorders [42]. Many studies have shown that HO-1 activation is an important response to the threat of oxidative stress to neurons as it can rescue neurons from oxidative stress and cell death [43, 44]. HO-1 has been strongly linked to reduced inflammation [45]. Similarly, HO-1 knockout mice have been shown to develop inflammatory disease and are sensitive to experimental sepsis [46], while the pharmacological activation of HO-1 has been found to be beneficial in the inflammation animal model [47]. Excessive glutamate that is triggered by oxidative stress or ROS accumulation induces neuronal apoptosis and cell death. This process is similar in nature to numerous neurodegenerative diseases and some pathological conditions, such as ischemia and trauma [3, 48, 49]. The literature has shown that glutamate can induce Nrf2 and HO-1 suppression, which is followed by the induction of inflammation and neurodegeneration in the postnatal brain as well as in cells. In the present study, we have demonstrated that a single dose of exogenously provided glutamate caused the induction of neuroinflammation and neurodegeneration in the postnatal rat brain. Additionally, glutamate treatment in SH-SY5Y and BV2 cells also induced the activation of proinflammatory and proapoptotic markers, such as NF-_*k*_B and caspase-3 expression. As per our results, these toxic effects of glutamate are AMPK dependent because our findings indicate that either compound C or siRNA-mediated silencing of AMPK can diminish the toxic effects of glutamate treatment both in vivo and in vitro.

Although some additional work is necessary to elucidate the mechanisms that are involved in this current network, the findings from this study have identified a major role of AMPK activation by anthocyanins in the attenuation of oxidative stress through the activation of Nrf2/HO-1 signaling. Furthermore, our study suggests that anthocyanins might be a pharmacological therapeutic agent for use in combination with other potential drugs in the treatment of various neurodegenerative diseases.

## Conclusions

Our data demonstrate that glutamate could induce its neurotoxicity within 2–3 h in AMPK-dependent manner in the developing rat’s brain. Similarly, we report for the first time that the ability of anthocyanins to reverse glutamate-suppressed Nrf2/HO-1 is AMPK dependent in young rats. This study demonstrates that anthocyanin is a highly potent agent in vivo and in vitro against glutamate-induced oxidative stress and neurodegeneration as it have attractive drug-like properties.

## Additional file

Additional file 1: Figure S1. Anthocyanin upregulated glutathione levels (GSH and GSH/GSSG ratio) against glutamate in the developing rat brain. The histograms show (a) the levels of total Glutathione (GSH) and (b) the ratio of GSH/GSSG in the brain homogenates of rat pups 4 h after glutamate and anthocyanin treatment. All the procedures were followed as provided by the manufacturer. These assays were performed in triplicate with the same results. Significance, ***P* < 0.001 and ^##^ *P* < 0.001, respectively. Figure S2d. (PDF 189 kb)

## Abbreviations

ALS: Amyotrophic lateral sclerosis
AMPK: 5′ adenosine monophosphate-activated protein kinase
CNS: Central nervous system
DCFH-DA: Dichloro-dihydro-fluorescein diacetate
DMEM: Dulbecco’s modified Eagle’s medium
DMSO: Dimethyl sulfoxide
FBS: Fetal bovine serum
FJB: Fluoro-Jade B
HO-1: Heme oxygenase-1
MTT: 3-(4,5-dimethylthiazol-2-yl)-2,5-diphenyltetrazolium bromide
Nrf2: Nuclear factor-E2-related factor 2
ROS: Reactive oxygen species
SD: Sprague-Dawley
SDS-PAGE: Sodium dodecyl sulfate polyacrylamide gel electrophoresis
TUNEL: Terminal deoxynucleotidyl transferase (TdT)-mediated dUTP nick-end labeling
