# Supplementary material for: Anthocyanins abrogate glutamate-induced AMPK activation, oxidative stress, neuroinflammation, and neurodegeneration in postnatal rat brain
Source: J Neuroinflammation. 2016 Nov 8;13:286. doi: 10.1186/s12974-016-0752-y (PMC5100309; doi:10.1186/s12974-016-0752-y)
Supplement: Additional file 1: Figure S1. — Anthocyanin upregulated glutathione levels (GSH and GSH/GSSG ratio) against glutamate in the developing rat brain. The histograms show (a) the levels of total Glutathione (GSH) and (b) the ratio of GSH/GSSG in the brain homogenates of rat pups 4 h after glutamate and anthocyanin treatment. All the procedures were followed as provided by the manufacturer. These assays were performed in triplicate with the same results. Significance, **P < 0.001 and ## P < 0.001, respectively. Figure S2d. (PDF 189 kb) [file 12974_2016_752_MOESM1_ESM.pdf]

Supplementary Materials

Treatment (Glu and Antho) detail *in vivo*

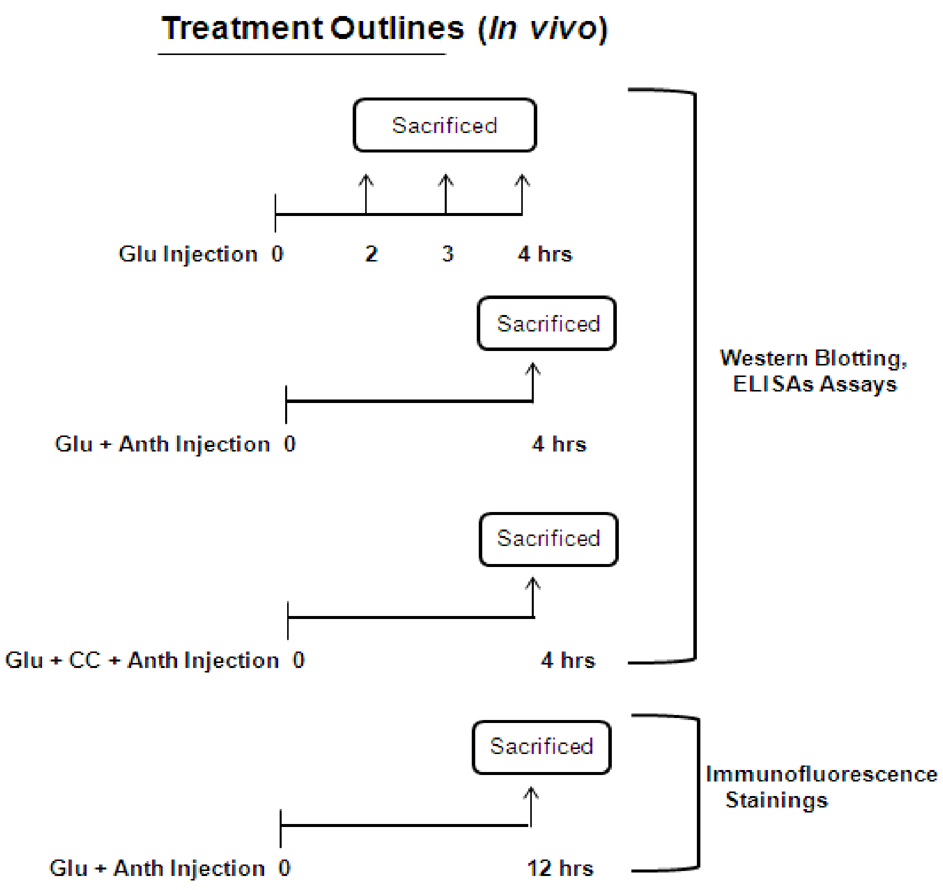

Fig. S1

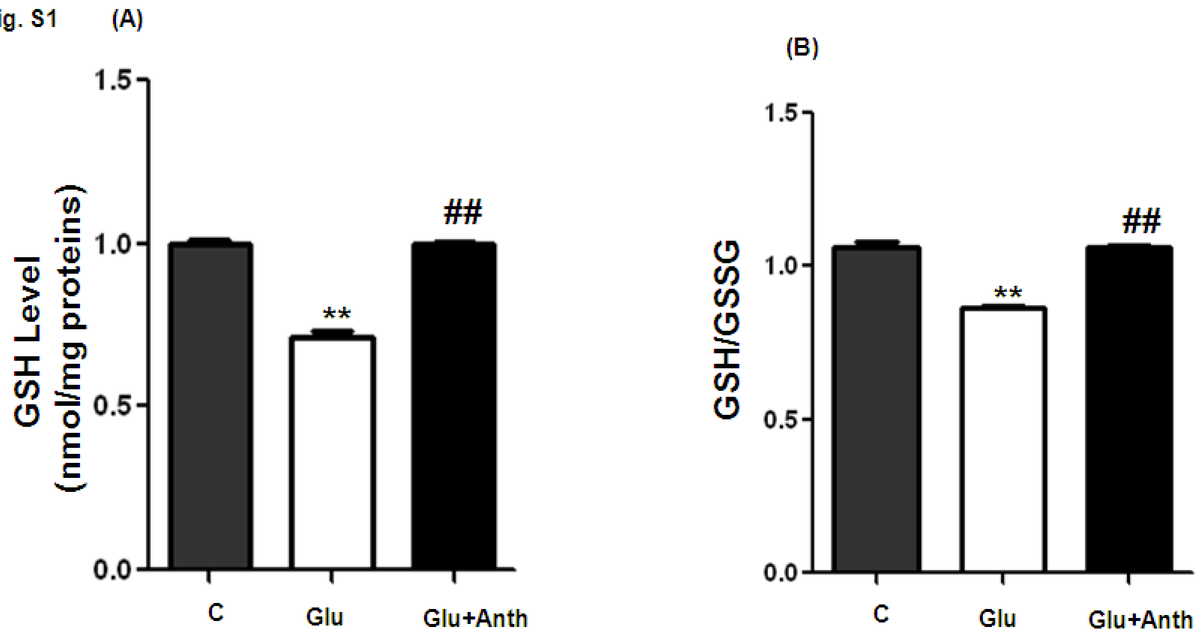

Suppl. Fig. 1: Anthocyanin upregulated glutathione levels (GSH and GSH/GSSG ratio) against glutamate in the developing rat brain. The histograms showing **(A)** the levels of total Glutathione (GSH) and **(B)** the ratio of GSH/GSSG in the brain homogenates of rat pups 4 h after glutamate and anthocyanin treatment. All the procedures were followed as provided by the manufacturer. These assays were performed in triplicate with the same results. Significance; $^{**}P<0.001$ and $^{##}P<0.001$, respectively.

Fig. 2d

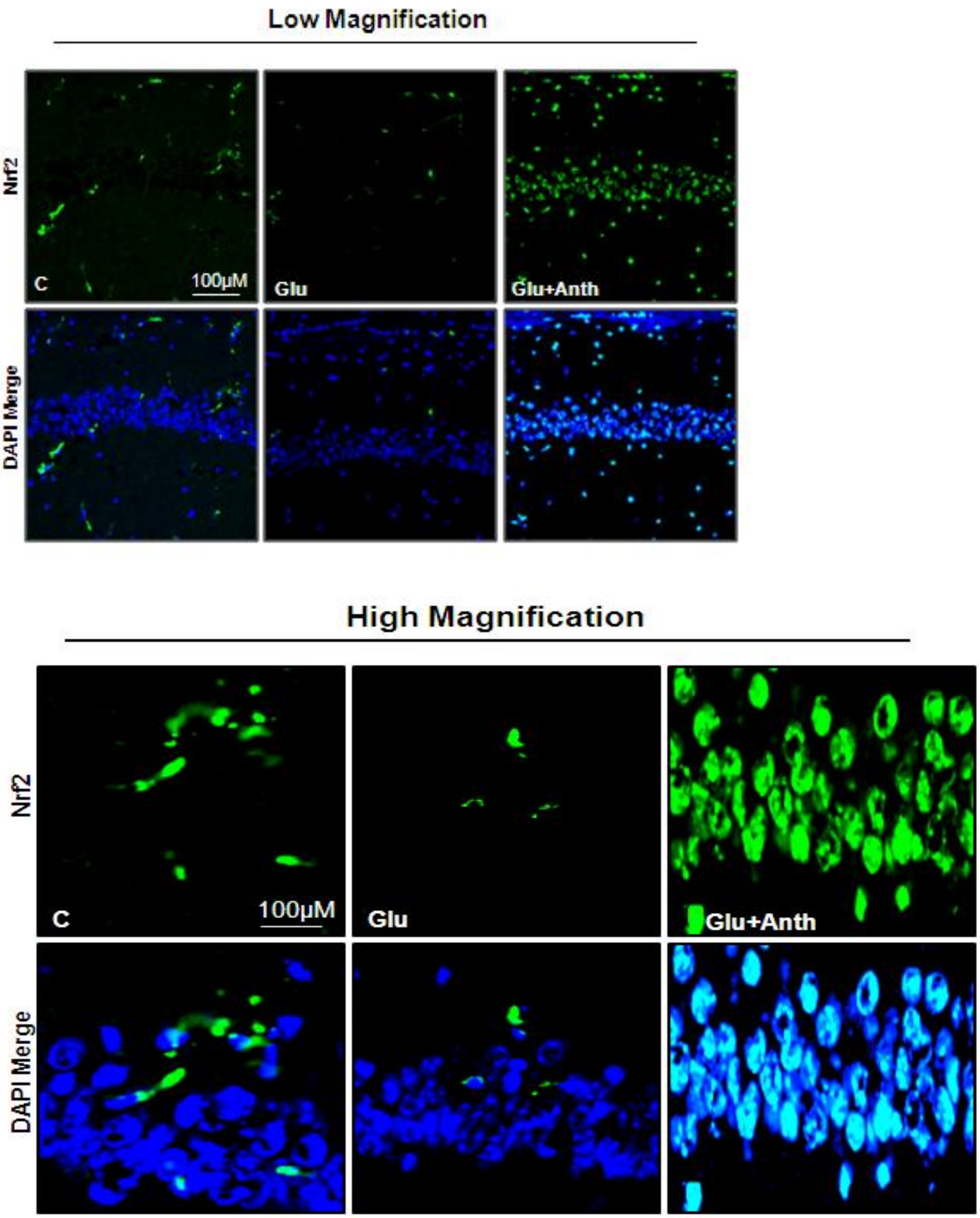
Low Magnification
Nrf2
DAPI Merge
C
100μM
Glu
Glu+Anth
High Magnification
Nrf2
DAPI Merge
C
100μM
Glu
Glu+Anth
